# Familial Congenital Hypothyroidism due to Compound Heterozygous Variants in the TPO Gene: Neonatal Diagnosis, Phenotypic Variability, and Lessons From Long‐Term Follow‐Up in Three Brothers

**DOI:** 10.1155/crig/8788438

**Published:** 2026-10-27

**Authors:** Mody Diop, Roosler Billy Telcide, Narcisse Elenga

**Affiliations:** ^1^ Clinical Genetics Unit Mother-Child Department CHU de Guyane Cayenne 97306 French Guiana France; ^2^ Department of Pediatrics CHU de Guyane Cayenne 97306 French Guiana France

**Keywords:** compound heterozygosity, congenital hypothyroidism, dyshormonogenesis, family screening, French Guiana, genetic counseling, levothyroxine monitoring, neonatal diagnosis, TPO

## Abstract

Dyshormonogenesis accounts for 15%–20% of permanent congenital hypothyroidism (CH), and pathogenic variants in the thyroid peroxidase (TPO) gene are its most frequent cause. We report three brothers, all diagnosed with severe CH in the neonatal period and referred to our Medical Genetics Unit in 2021 for molecular characterization and genetic counseling. Whole‐exome sequencing, followed by a targeted thyroid gene panel, identified the same compound heterozygous TPO genotype in all three: NM_000547.6:c.1297G > A, p.(Val433Met), inherited from the carrier mother, and NM_000547.6:c.1964G > C, p.(Cys655Ser), inherited from the carrier father, confirming autosomal recessive dyshormonogenetic CH. At neonatal diagnosis, initial TSH exceeded 100 mIU/L in all three brothers (reference range approximately 1–20 mIU/L beyond the first days of life (Wassner 2018 and Léger et al. 2014)). Despite this identical genetic cause and comparable neonatal severity, the three brothers (hereafter Patient 1, Patient 2, and Patient 3, the index case) went on to show markedly divergent clinical trajectories. Patient 1 was treated continuously from birth. Patient 2 was correctly identified neonatally through a proactive family thyroid screening, prompted by his elder brother’s pre‐existing diagnosis, but was then lost to adequate endocrine follow‐up: his levothyroxine dose stayed static and unadjusted for several years while his TSH remained persistently elevated. Patient 3, the index case, was diagnosed at Day 3 through Guthrie neonatal screening, and it was his diagnosis that prompted the family‐wide reassessment which ultimately confirmed the genotype in all three brothers. He additionally carries a heterozygous SLC26A4 variant of uncertain significance. Together, these three cases show that the prognosis of treated neonatal CH depends as much on the quality of postdiagnosis monitoring as on how quickly the initial diagnosis is made. They also make the case for systematic family thyroid screening once a sibling is known to carry dyshormonogenetic CH, and for the value molecular diagnosis adds to parental counseling and reproductive planning.

## 1. Introduction

Congenital hypothyroidism (CH) is the most common neonatal endocrine disorder, affecting approximately 1 in 2000–4000 live births [1]. It results from insufficient thyroid hormone production, which is essential for neurological development, myelination, and somatic growth during the neonatal period and early childhood. Left untreated, or treated too late, CH causes irreversible neurocognitive impairment; universal Guthrie neonatal screening programs have largely prevented this outcome when diagnosis and treatment are timely [2]. Yet a good long‐term outcome depends on more than an early diagnosis: it equally requires sustained, well‐monitored levothyroxine dose titration throughout the critical developmental years of childhood.

Etiologically, CH divides into thyroid dysgenesis (80%–85% of the cases) and dyshormonogenesis (15%–20%); the latter arises from inherited, autosomal recessive defects in thyroid hormone biosynthesis [2, 3]. The genes most often implicated include thyroglobulin (TG), the sodium‐iodide symporter (SLC5A5), pendrin (SLC26A4), the dual oxidases (DUOX1 and DUOX2), and thyroid peroxidase (TPO) [3, 4]. TPO encodes a membrane‐bound heme enzyme required for iodide organification and for coupling iodotyrosines into thyroxine; biallelic loss‐of‐function variants abolish this function, causing complete iodide organification defect (TIOD) and permanent severe CH [5, 6]. Whole‐exome sequencing (WES) has considerably widened the diagnostic scope in this condition. It now routinely identifies compound heterozygous genotypes, with each variant inherited in trans from a carrier parent, and can also flag potential oligogenic modifiers such as SLC26A4 [7].

We report on three brothers, all diagnosed with severe CH in the neonatal period and followed by the pediatric endocrinology team at the Centre Hospitalier de Cayenne (CHC) in French Guiana. The family was referred to our Medical Genetics Unit in 2021 for molecular characterization and genetic counseling. By the time of referral, the clinical diagnosis of familial dyshormonogenetic CH had already been made in all three children, so our role was to identify the causative variant, confirm parental carrier status, and give the couple precise reproductive counseling. What makes this family instructive is the divergence in their long‐term outcomes despite an identical genotype and equally severe neonatal hypothyroidism: it offers a clear clinical lesson on what actually determines prognosis in treated CH.

## 2. Case Presentation

### 2.1. Clinical Context and Referral

The three brothers were born to a nonconsanguineous couple and have been followed since birth by the pediatric endocrinology team at the CHC, led by the referring pediatric endocrinologist. Three consecutive male siblings with severe CH raised clinical suspicion of a hereditary dyshormonogenetic etiology, and in 2021, the referring physician sent the family to our Medical Genetics Unit for molecular diagnosis and genetic counseling. Figure 1 shows the family pedigree, and Table 1 summarizes the main clinical, biological, and genetic findings in the three brothers.

**FIGURE 1:**
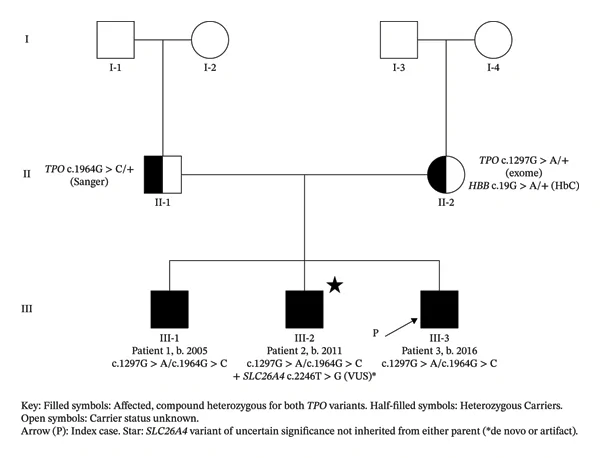
Family pedigree. Generation I: Paternal and maternal grandparents; carrier status unknown. Generation II: Father, heterozygous for TPO NM_000547.6:c.1964G > C, p.(Cys655Ser) (Class 4, Sanger confirmed); mother, heterozygous for TPO NM_000547.6:c.1297G > A, p.(Val433Met) (Class 4, exome confirmed); incidental finding in the mother: *HBB* c.19G > A (p.Glu7Lys), hemoglobin C heterozygosity, not clinically significant (absent in Patient 1). Generation III: Patient 1 (III‐1, b. 2005), Patient 2 (III‐2, b. 2011; star = additional SLC26A4 VUS, not inherited from either parent), Patient 3 (III‐3, b. 2016; arrow = index case, i.e., the sibling whose diagnosis triggered family‐wide reassessment—distinct from Patient 1, who underwent the exome duo), all compound heterozygous for both TPO variants (filled squares = affected). Half‐filled symbols represent heterozygous carrier parents. Pedigree follows ACMG/NSGC standard conventions.

**TABLE 1:** Summary of clinical, biological, and genetic findings in the three brothers.

	Patient 1	Patient 2	Patient 3 (index)
Date of birth	19 Jan 2005	21 Aug 2011	26 Oct 2016
Gestational age	35 weeks	Term	33 weeks
Birth weight, length	2890 g, 48 cm	3120 g, 51 cm	3030 g, 47 cm
Neonatal TSH (mIU/L)	> 100	> 100	> 100
Reference range (TSH/FT4/FT3)^‡^	∼1–20/11–28/3–9	∼1–20/11–28/3–9	∼1–20/11–28/3–9
Latest TSH (Oct 2023)	2.83 mIU/L	4.54 mIU/L	3.30 mIU/L
Current LT4 dose	125 μg/day	100 μg/day	65 μg/day
Midparental target height	Not calculable^∗^	Not calculable^∗^	Not calculable^∗^
Thyroid palpation	Not separately documented^†^	Not separately documented^†^	Not separately documented^†^
Goiter on ultrasound	No (normal gland)	Yes, 13 mL (compensatory)	Yes, 13 mL (heterogeneous)
Cognitive assessment method	Global clinical evaluation, CAMSP/MDPH	Global clinical evaluation, CAMSP/MDPH	Global clinical evaluation, CAMSP/MDPH
*TPO* variant 1 (maternal)	c.1297G > A p.(Val433Met) het. Class.4	Same	Same
*TPO* variant 2 (paternal)	c.1964G > C p.(Cys655Ser) het. Class.4	het. Class.3‐4	het. Class.3
Additional variant	—	*SLC26A4* c.2246T > G het. Class.3 (de novo/artifact)	—

^∗^Parental heights were not systematically documented in the retrievable medical record.

^†^Thyroid palpation findings were not separately recorded in the clinical notes beyond the general physical examination (unremarkable in all three); thyroid ultrasound served as the objective morphological assessment. Extended family history of thyroid disease was specifically investigated and was negative.

^‡^Reference ranges reflect commonly reported age‐appropriate values for the neonatal/early infancy period (mIU/L for TSH; pmol/L for FT4/FT3) [8, 9], as the original assay‐specific reference ranges from 2005/2011/2016 could not be retrieved from the archived laboratory reports.

Molecular investigations were carried out over the following months: an exome duo for Patient 1 and his mother (Biomnis–Eurofins, Lyon, November 2023, funded by the Centre Hospitalier Universitaire de Guyane), followed by a targeted thyroid gene panel for Patients 2 and 3 and Sanger segregation analysis of the father (AP‐HP BioGeM Laboratory, Paris, October 2024; Biomnis, January 2025). The results confirmed compound heterozygous pathogenic TPO variants in all three brothers, each allele traced to its respective carrier parent (A note on terminology: the original submission referred to the exome‐tested sibling as “the index case.” We clarify here that the exome duo was performed on Patient 1, while Patient 3 is the clinical index case whose diagnosis triggered the family‐wide reassessment; these are two distinct roles, now disambiguated throughout the text).

### 2.2. Patient 1 (Born 19 January 2005, 35 Weeks of Gestation)

Patient 1 was born prematurely at 35 weeks of gestation (no obstetric cause of prematurity was identified; birth weight 2890 g, length 48 cm). Maternal thyroid function was not specifically investigated during this pregnancy, and no clinical signs suggestive of maternal thyroid dysfunction were reported. Generalized hypotonia and poor weight gain in the immediate neonatal period prompted urgent thyroid function testing, which revealed severe primary hypothyroidism: TSH > 100 mIU/L, T4L 18.8 pmol/L, and T3L 5.5 pmol/L (reference range for the immediate neonatal period: TSH approximately 1–20 mIU/L beyond the first days of life, following the physiological neonatal TSH surge which can reach up to 70 mIU/L within the first 24 h before rapidly declining [8]; free T4 approximately 11–28 pmol/L; and free T3 approximately 3–9 pmol/L, with minor variation across assay platforms [8, 9]). Anti‐TG and anti‐TPO antibodies were negative. Physical examination was, otherwise, unremarkable (no dysmorphism, normal cardiorespiratory and abdominal examination); thyroid palpation was not separately documented in the clinical notes; thyroid ultrasound, reported in the following, served as the objective morphological assessment. Levothyroxine (Levothyrox®) was initiated in the neonatal period at 45 μg/day and has been continued uninterrupted since birth. After progressive uptitration in response to growth and biological monitoring, the current dose is 125 μg/day.

Despite early and continuous treatment, Patient 1 has had learning difficulties requiring educational support throughout his schooling (assessed by global clinical evaluation, with referral to the regional early intervention/disability services [CAMSP/MDPH]); these are attributable in part to the neurological impact of prematurity and of severe in utero hypothyroidism before postnatal treatment began [10]. On clinical review, he is behaviorally normal, with no dysmorphism, and clinically euthyroid. His TSH has fluctuated between 2.83 mIU/L (October 2023, on 125 μg/day) and 17.5 mIU/L (May 2023) over the documented follow‐up period, which reflects how hard it can be to keep TSH stable through adolescence. Thyroid ultrasound was structurally normal in September 2012 (right lobe 30 × 11 × 8 mm, left lobe 29 × 11.8 × 8.5 mm, and isthmus 2 mm) and remained normal in May 2023. Bone age in June 2023 was at least 19 years, concordant with his chronological age.

### 2.3. Patient 2 (Born 21 August 2011, at Term): A Confirmed Neonatal Diagnosis Left Without Adequate Follow‐Up

Patient 2 was born at term (birth weight 3120 g, length 51 cm), with no overt neonatal symptoms. Because his brother Patient 1 had already been diagnosed with CH at birth, and a hereditary dyshormonogenetic etiology was already suspected, a systematic thyroid function workup was proactively requested for him shortly after birth. This family‐targeted screening, done at 16 days of age (6 September 2011), revealed severe primary hypothyroidism: TSH > 100 mIU/L, T4L 8.17 pmol/L, and T3L 5.9 pmol/L. Anti‐TG and anti‐TPO antibodies were negative. Thyroid ultrasound on 29 September 2011 showed a morphologically normal gland (right lobe: H 20 mm, W 13 mm; left lobe: H 19 mm, W 9 mm; isthmus 6 mm; and homogeneous echotexture without nodule).

The neonatal biochemical diagnosis was thus correctly established at 16 days of life, a direct benefit of the systematic family approach. What followed is the most instructive part of this family’s story, and the most concerning. Levothyroxine was prescribed at 50 μg/day, but Patient 2 was not subsequently placed under regular, structured endocrine supervision (his diagnosis came from a family‐targeted clinical and biological workup rather than the national Guthrie screening circuit, so it was not exposed to that pathway’s sample‐transport vulnerabilities; no specific administrative or clinical cause for the subsequent loss to follow‐up could be identified retrospectively from the available records). His levothyroxine dose stayed at 50 μg/day for years afterward, regardless of his weight gain or the persistently elevated TSH seen on the few controls that were obtained: 6.5 mIU/L in April 2012, 17.5 mIU/L in July 2013, and 8.0 mIU/L in February 2014. All of these values sit well above the European consensus target of ≤ 2 mIU/L for children under 3 [11], yet none prompted a dose adjustment. He was effectively lost to adequate endocrine follow‐up throughout his preschool years and into the start of primary school.

Serial height and weight measurements from this period were not systematically recorded in the retrievable medical record, so we cannot formally document a discrete growth deceleration or catchup phase; the persistently elevated TSH values above remain the main biochemical evidence of this undertreatment window. Parental heights were likewise not systematically documented, which precludes calculating a midparental target height for the three brothers. None of the three, however, showed growth substantially outside the expected population percentiles at last review (see the following).

It was only in November 2016, when Patient 2 was five years old, that the family was properly re‐evaluated at the CHC, triggered by Patient 3’s diagnosis as the index case. He measured 104 cm at this re‐evaluation. At that point, the referring physician systematically reassessed all three brothers. Patient 2’s dose was progressively increased: 50 μg/day until 2016, then 88 μg/day in 2017, and 100 μg/day currently. At clinical review on 9 December 2024, he weighed 53 kg (71st percentile) and measured 160 cm (71st percentile), clinically euthyroid on 100 μg/day with TSH 4.54 mIU/L in October 2023. He has, however, needed consistent educational support throughout his school career for significant concentration difficulties and learning disabilities, sequelae of the years of chronic undertreatment during a period of peak neurodevelopmental vulnerability.

Thyroid ultrasound in May 2023 revealed bilateral thyroid enlargement: right lobe 25 × 41 × 15 mm (volume 8 cc), left lobe 19 × 40 × 12 mm (volume 5 cc), isthmus 4 mm, and homogeneous echotexture; this is a compensatory goiter, consistent with years of TSH hyperstimulation (total volume 13 mL at age 11 years 9 months, well above the age‐expected range of approximately 2.7–4 mL for the prepubertal/early pubertal period reported in published pediatric normative ultrasound data [12]). Bone age in June 2023 was 10 years, appropriate for his chronological age. Molecular analysis (AP‐HP thyroid gene panel, October 2024) identified the same compound heterozygous TPO variants as his brothers, plus a heterozygous SLC26A4 variant: NM_000441.2:c.2246T > G, p.(Ile749Ser), Class 3. Paternal Sanger sequencing confirmed this allele was absent in the father, and subsequent maternal testing confirmed it was absent in the mother too, so it was inherited from neither parent. This could reflect a de novo occurrence or a technical/sequencing artifact; we treat it as a limitation of this finding.

### 2.4. Patient 3 (Born 26 October 2016, 33 weeks of Gestation): The Index Case

Patient 3 was born prematurely at 33 weeks of gestation (no obstetric cause of prematurity was identified). The specific method used to establish gestational age (early dating ultrasound versus last menstrual period) is not specified in the retrievable obstetric record. Maternal thyroid function was not specifically investigated during this pregnancy, and no clinical signs suggestive of maternal thyroid dysfunction were reported. Birth weight (3030 g) was at the upper range for 33 weeks of gestation, consistent with a large‐for‐gestational‐age but genuinely preterm infant; a high birth weight does not, in itself, call gestational age into question when the latter is established independently by obstetric dating rather than by weight‐based estimation. (length 47 cm and head circumference 35 cm). CH was detected at Day 3 of life through the systematic Guthrie neonatal screening program, in a context of hypotonia and feeding difficulties. Confirmatory thyroid function testing showed severe primary hypothyroidism: TSH > 100 mIU/L, T4L 18.8 pmol/L (reference 19–45 pmol/L), and T3L 5.5 pmol/L. Anti‐TG and anti‐TPO antibodies were negative. Levothyroxine was started in November 2016 at 45 μg/day; the current dose is 65 μg/day.

Patient 3’s diagnosis at Day 3 was what set the comprehensive family re‐evaluation in motion. The referring physician immediately reassessed all three brothers, corrected Patient 2’s management, and referred the family to genetics, which ultimately led to the molecular characterization described here. In that sense, the Guthrie screening program did not just benefit Patient 3 directly; it also, retrospectively, benefited Patient 2, whose years of chronic undertreatment were finally corrected.

At clinical review on 9 December 2024, Patient 3 weighed 23.7 kg (28th percentile) and measured 128 cm (47th percentile). He was clinically euthyroid on 65 μg/day with TSH 3.30 mIU/L in October 2023, the best‐controlled TSH of the three brothers at a comparable age. Mild learning difficulties were noted (assessed by global clinical evaluation, with referral to the regional early intervention/disability services [CAMSP/MDPH]). Thyroid ultrasound in May 2023 revealed an enlarged, heterogeneous, hypervascularized gland (right lobe: 22 × 39 × 15 mm, volume 7 cc; left lobe: 24 × 34 × 14 mm, volume 6 cc; isthmus 2 mm; reactive bilateral cervical lymphadenopathy, largest node 7 mm in right IIa region) (total volume 13 mL at age 6 years 7 months, several‐fold above the age‐expected range of approximately 1.5–2.5 mL for this age reported in published pediatric normative ultrasound data [12]). The ultrasound pattern initially raised the question of autoimmune thyroiditis, but the persistently negative thyroid antibodies argue against it; follicular hyperplasia secondary to residual organification defect seems the more likely explanation. Bone age in June 2023 was 5 years, appropriate for his age.

### 2.5. Molecular Genetic Analysis

We performed exome duo sequencing for Patient 1 and his mother in November 2023 (Biomnis–Eurofins, Lyon; Human Exome 2.0 Plus Comprehensive Exome Spike‐In capture; Illumina NovaSeq 6000, paired‐end 2 × 150 bp; 53 million read pairs; 98% bases covered at > 10×; SNV/indel analysis via BiomniSeq4/GATK4; variant annotation via GermlineVar, SeqOne, Montpellier). Patients 2 and 3 then underwent a targeted thyroid gene panel in October 2024 (18 genes: DUOX2, DUOXA2, HES1, NKX2‐5, IGSF1, IYD, NKX2‐1, PAX8, SLC26A4, SLC5A5, TG, TPO, TRH, TSHB, TSHR, TBLX1, FOXE1; SureSelectV11, Illumina; AP‐HP BioGeM Laboratory, Hôpital Robert Debré, Paris), and paternal Sanger segregation analysis followed in January 2025 (Biomnis, Lyon). All variant frequencies below refer to gnomAD v4.1.0 (GRCh38, cross‐referenced via VarSome).

Two compound heterozygous TPO variants were confirmed in all three brothers. NM_000547.6:c.1297G > A, p.(Val433Met) (exon 8, Chr2:g.1481335 GRCh37; gnomAD frequency 0.008%; reported pathogenic, PMID 12938097; REVEL deleterious) was heterozygous in all three children and inherited from the carrier mother (Class 4 at both laboratories) [13]. NM_000547.6:c.1964G > C, p.(Cys655Ser) (exon 11, Chr2:g.1497769 GRCh37/chr2:1493997 GRCh38) is absent from ClinVar and appears only as an ultrarare singleton allele in gnomAD v4.1.0 (exome frequency 0.000000684, absent from genomes); it is highly conserved (phyloP100 = 9.168), carries a pathogenic‐leaning aggregated in silico consensus (PP3), and is functionally analogous to the pathogenic p.Cys655Phe (PMID 32908504) [14]. This variant was heterozygous in all three brothers and inherited from the carrier father (Class 4 at Biomnis and Class 3 at AP‐HP, reflecting differing laboratory thresholds). Sanger sequencing of the father confirmed heterozygous c.1964G > C and absence of c.1297G > A, establishing the trans configuration. In Patient 2 only, the panel additionally identified the SLC26A4 variant NM_000441.2:c.2246T > G, p.(Ile749Ser) (chromosome 7, exon 20; an ultrarare singleton allele in gnomAD v4.1.0, exome frequency 0.000000709, absent from genomes; moderate conservation, phyloP100 = 5.11; benign‐leaning aggregated in silico consensus, BP4; class 3), which was absent in the father. Table 2 presents the detailed in silico scores for both variants.

**TABLE 2:** In silico pathogenicity assessment of the two novel/ultrarare variants (VarSome, hg38/GRCh38, gnomAD v4.1.0).

	*TPO* c.1964G > C, p.(Cys655Ser)	*SLC26A4* c.2246T > G, p.(Ile749Ser)
gnomAD exomes	*f* = 0.000000684 (cov. 62.6x)	*f* = 0.000000709 (cov. 23.9x)
gnomAD genomes	Not found (cov. 32.4x)	Not found (cov. 31.2x)
Conservation (phyloP100way)	9.168 (highly conserved)	5.11 (moderately conserved)
Aggregated in silico consensus (ACMG criterion)	PP3—Pathogenic moderate (4/5 tools pathogenic)	BP4—Supporting (benign‐leaning)

Frequencies, conservation scores, and aggregated in silico consensus were retrieved from VarSome (gnomAD v4.1.0, GRCh38). Both variants remain absent from gnomAD genomes and are present only as ultrarare/singleton alleles in exomes, consistent with recent‐origin, disease‐associated variants. The TPO variant shows a strongly conserved residue (phyloP100 = 9.168) and a pathogenic‐leaning in silico consensus (PP3), supporting its causal role; the SLC26A4 variant shows a benign‐leaning consensus (BP4) and lower conservation, consistent with its provisional classification as a variant of uncertain significance/possible modifier rather than a primary causal allele.

Sanger sequencing traces and NGS read visualizations (IGV) for the reported variants have been requested from the performing laboratories (Biomnis‐Eurofins and AP‐HP BioGeM) and will be provided as supplementary data upon receipt. The certified molecular genetics reports issued by these laboratories, already cited above, constitute the diagnostic documentation of record for the variants reported in each family member.

## 3. Discussion

### 3.1. The Molecular Architecture of the Compound Heterozygous TPO Genotype

TPO encodes thyroid peroxidase, the apical membrane enzyme in thyroid follicular cells that oxidizes iodide and couples iodotyrosine residues to form T4 and T3. When biallelic loss‐of‐function TPO variants abolish this activity, the result is complete iodide organification defect (TIOD), identifiable by a positive perchlorate discharge test. TPO variants are collectively the most common molecular cause of dyshormonogenetic CH; more than 100 distinct disease‐associated alleles have been described [5, 6].

The maternally inherited variant c.1297G > A, p.(Val433Met) has already been reported as pathogenic, with an associated positive perchlorate discharge test, in published kindreds (PMID 12938097) [13]. It replaces a conserved valine residue in the peroxidase domain with methionine, which in silico prediction suggests disrupts protein folding (REVEL: damaging). The paternally inherited variant c.1964G > C, p.(Cys655Ser) has not previously been reported. Cysteine 655 is predicted to take part in a conserved disulfide bond critical to the tertiary structure of the peroxidase active site, a mechanism already documented for the adjacent p.Cys655Phe allele (PMID 32908504) [14]; this residue is strongly conserved (phyloP100 = 9.168) and carries a pathogenic‐leaning aggregated in silico consensus (PP3, VarSome/gnomAD v4.1.0), which further supports a causal role (Table 2). The two laboratories classified c.1964G > C differently (Class 4 at Biomnis versus Class 3 at AP‐HP), which reflects differing interpretive thresholds for novel missense variants rather than genuine disagreement about pathogenicity; we think the convergent clinical picture, three severely affected compound heterozygous brothers with the parental allele‐in‐trans confirmed, amounts to robust functional evidence for pathogenicity. We recommend reclassifying c.1964G > C as Class 4 in the context of this family.

### 3.2. Three Brothers, One Genotype, Three Outcomes: The Decisive Role of Postdiagnosis Management

What this case series mainly shows is that, in dyshormonogenetic CH, long‐term neurodevelopmental prognosis does not depend on the genotype, or even solely on the neonatal TSH level; it depends on the quality of biological monitoring and how regularly levothyroxine doses are adjusted throughout childhood. All three brothers had TSH above 100 mIU/L at neonatal diagnosis, yet by school age, their outcomes differed markedly.

Patient 1, treated continuously from birth with regular dose uptitration (30 μg at 4 years, 125 μg currently), has the mildest cognitive profile of the three; his learning difficulties are mild and largely explained by his prematurity. Patient 3, diagnosed at Day 3 with immediate treatment and structured follow‐up from November 2016, already has the best TSH control of the three at a comparable age (3.30 mIU/L at age 7 on 65 μg/day). Patient 2 is the exception, and the instructive one: although he was correctly identified neonatally at Day 16 through family screening, he spent roughly 5 years on a static, unadjusted 50 μg/day dose, with TSH values of 17.5 mIU/L (July 2013) and 8.0 mIU/L (February 2014) going unaddressed. He now has the most pronounced residual cognitive impairment of the three siblings, along with a bilateral compensatory goiter on ultrasound, a direct morphological trace of years of TSH hyperstimulation.

This fits with published evidence that cumulative TSH burden during the first 3 years of life is the strongest modifiable predictor of neurocognitive outcome in treated CH, regardless of etiology or initial TSH at diagnosis [1, 10, 11]. European consensus guidelines recommend keeping TSH below 2 mIU/L in the first year, with dose adjustment every 3 months during infancy [11]. Patient 2 shows what happens when these recommendations are not followed, not because his diagnosis was missed, but because an early, correct neonatal diagnosis was never backed by a proper monitoring and dose‐adjustment system.

### 3.3. The Dual Lesson of Family Screening: It Works—But It Is Not Enough

Patient 2’s story captures a paradox with direct public health implications. The systematic family thyroid screening performed at day 16, justified by Patient 1’s pre‐existing diagnosis, was clinically appropriate and biochemically conclusive: it identified severe hypothyroidism 2 weeks after birth in an, otherwise, asymptomatic term neonate, someone standard Guthrie screening alone would not have caught. That is exactly the kind of proactive, family‐based approach recommended once a sibling is known to carry an autosomal recessive thyroid disorder.

Yet this diagnostic success never translated into therapeutic benefit because the downstream management chain failed. This raises a real question for pediatric practice in French Guiana: once a systematic family thyroid screening identifies a severely hypothyroid neonate, what protocol ensures structured, longitudinal endocrine follow‐up with regular dose adjustment, whether the diagnosis came through national screening or targeted family testing? Transport disruptions in the national Guthrie screening circuit (samples are shipped to the mainland reference laboratory at CHU de Lille for analysis) are a recognized, recurrent vulnerability for delayed diagnosis, but they do not explain Patient 2’s case: his diagnosis came directly from family‐targeted testing, not through that national circuit. What his loss to follow‐up reflects, in our view, is a different and more general vulnerability: the absence, at the time, of a formalized pathway automatically linking any positive result; however, it was obtained, to structured, recallable endocrine follow‐up. No specific administrative cause for this gap could be established retrospectively. A formal CH monitoring pathway, with structured recalls, dose adjustment algorithms tied to weight and age, and telemedicine links to mainland French reference centers would help prevent this kind of avoidable outcome in future cases.

### 3.4. SLC26A4 Variant in Patient 2: Modifier or Incidental Finding?

Patient 2 also carries the SLC26A4 variant c.2246T > G, p.(Ile749Ser), classified as a VUS (Class 3) and absent in both parents on segregation testing. SLC26A4 encodes pendrin, an anion transporter involved in thyroid iodide efflux, and heterozygous SLC26A4 variants have been proposed as potential modifiers of thyroid phenotype alongside TPO mutations [15]. Its absence in both parents could mean a de novo event, or it could reflect a technical or sequencing artifact; we treat this as an unresolved limitation of the present report rather than evidence for either explanation. For now, the best explanation for Patient 2’s more severe clinical and morphological phenotype remains his years of inadequate levothyroxine management, not any oligogenic contribution. Functional characterization would be needed to reassess how relevant this VUS actually is.

### 3.5. Shared Cognitive Phenotype Across the Three Brothers

Beyond the TPO and SLC26A4 variants already discussed, the exome duo analysis of Patient 1 and his mother did not turn up any other variant that could plausibly explain the learning difficulties shared by the three brothers. Given how closely, within this family, the quality of thyroid hormone control tracks the severity of cognitive sequelae (mildest in Patient 3 and Patient 1, most pronounced in Patient 2), we think the simplest explanation remains the direct neurodevelopmental impact of severe hypothyroidism, antenatal and neonatal in origin and worsened by Patient 2’s prolonged undertreatment, rather than some separate, unidentified genetic cause.

### 3.6. Added Value of Molecular Diagnosis for This Family

The 2021 referral to our Medical Genetics Unit, and the molecular characterization that followed, added three concrete things beyond the clinical diagnosis that was already in place. It made targeted, highly accurate prenatal molecular diagnosis or preimplantation genetic testing (PGT‐M) possible for any future pregnancy, now that the precise compound heterozygous TPO genotype and parental carrier status are confirmed. It also opens the door to cascade genetic testing among the siblings of each parent since each allele has been traced to its respective carrier, which could identify further carriers in the extended family. And the detection of the novel c.1964G > C allele, together with its provisional reclassification to Class 4 based on this family’s clinical picture, feeds directly back into the broader evidence base for interpreting TPO variants.

### 3.7. Managing Familial CH in French Guiana: Structural Challenges and Necessary Responses

This case series comes out of a healthcare context with real structural challenges for managing chronic pediatric endocrine disorders. French Guiana’s geographic extent, its population’s mobility, the limited density of local specialists, and its cultural and linguistic diversity all make follow‐up gaps like the one Patient 2 experienced more likely here than in mainland France. The Guthrie neonatal screening program itself works well as Patient 3’s timely diagnosis shows, but the chain of monitoring after diagnosis remains a weak point. What we take from this is a case for dedicated familial CH protocols adapted to the local context: Systematic sibling screening whenever a hereditary etiology is suspected, structured biological recall systems to prevent loss to follow‐up, and telemedicine consultations to keep specialist‐level endocrine supervision available to families in geographically isolated communities.

## 4. Conclusion

We report three brothers with familial autosomal recessive CH caused by compound heterozygous TPO variants (c.1297G > A, p.Val433Met, maternal; c.1964G > C, p.Cys655Ser, paternal), all identified in the neonatal period with TSH above 100 mIU/L. Their genotype is identical and their neonatal hypothyroidism was comparably severe, so neither explains why their outcomes at school age diverged so sharply. What explains it is whether, after the neonatal diagnosis, levothyroxine was properly adjusted and biologically monitored through the critical years of neurodevelopment. Patient 2 makes the point clearly: Correctly identified neonatally through proactive family screening, then left without adequate management for several years, he shows that a correct diagnosis is necessary but not sufficient for a good outcome in treated CH. The molecular characterization that followed our Medical Genetics Unit referral in 2021 confirmed the causal genotype, established parental carrier status, and opened the way to precise reproductive counseling and prenatal testing for future pregnancies.

## Author Contributions

Mody Diop: conceptualization, molecular genetic analysis and interpretation, genetic counseling, writing–original draft, and writing–review and editing. Roosler Billy Telcide: clinical care and long‐term endocrine follow‐up of all three patients, acquisition of clinical data, and writing–review and editing. Narcisse Elenga: supervision and critical revision of the manuscript.

## Funding

This case series received no specific external research funding and was performed as part of the routine clinical activities of the Cayenne Hospital Center, French Guiana, France. The exome duo sequencing was funded through the routine clinical/diagnostic budget of the Centre Hospitalier Universitaire de Guyane.

## Disclosure

All authors have read and approved the final manuscript.

## Ethics Statement

This case series is based on retrospective, standard‐of‐care clinical and molecular data for a single family. Under French regulatory practice governing the secondary use of care data for research and publication (CNIL reference methodology MR‐004), patients and families followed in our department are informed, through departmental information notices, that their clinical data may be used for research and publication, with a right to object; this family raised no objection. This report is not an interventional or noninterventional research protocol under the French “Loi Jardé” and so does not fall within the scope of a Comité de Protection des Personnes (CPP). Beyond this standard legal basis, we also obtained written informed parental consent specifically for publication of this case report, including the clinical data and family pedigree, for all three patients; it is available upon request.

## Conflicts of Interest

The authors declare no conflicts of interest.

## Data Availability

The data supporting the findings of this case series are available from the corresponding author upon reasonable request. All patient data have been anonymized in accordance with applicable data protection regulations.
